# Molecular incidence and clearance of *Plasmodium falciparum* infection

**DOI:** 10.1186/s12936-015-0941-7

**Published:** 2015-10-22

**Authors:** Donald J. Krogstad, Ousmane A. Koita, Mouctar Diallo, John L. Gerone, Belco Poudiougou, Mahamadou Diakité, Yéya T. Touré

**Affiliations:** Center for Infectious Diseases, Tulane University School of Public Health and Tropical Medicine, #8317, J. Bennett Johnston Building, Room 510, 1324 Tulane Avenue, New Orleans, LA 70112 USA; Department of Tropical Medicine, Tulane University School of Public Health and Tropical Medicine, New Orleans, LA USA; Faculty of Medicine, Pharmacy and Odontostomatology, Mali-Tulane Tropical Medicine Research Center, University of Bamako, Bamako, Mali; Department of Epidemiology, Tulane University School of Public Health and Tropical Medicine, New Orleans, LA USA

**Keywords:** Clearance, Diagnosis, Epidemiology, Incidence, Malaria, Microscopy, Molecular methods, Prevalence, *Plasmodium falciparum*

## Abstract

**Background:**

Although the epidemiology of malaria has been based primarily on microscopy and rapid diagnostic tests, molecular methods are necessary to understand the complexity of natural infection in regions where transmission is intense and simultaneous infection with multiple parasite genotypes is common such as sub-Saharan Africa.

**Methods:**

To compare microscopic and molecular estimates of the incidence and clearance of *Plasmodium falciparum* infection, we followed 80 children monthly for 1 year in the village of Bancoumana in Mali.

**Results and discussion:**

Similar seasonal patterns were observed with both methods (rainy season peak, dry season nadir), although molecular methods detected more infections than microscopy (571 vs 331 in 906 specimens), more new infections (311 vs 104 during 829 person-months) and spontaneous clearance events (317 vs 116) and found higher incidence (0.38 vs 0.13 new genotypes/person/month, *p* < 0.001) and spontaneous clearance rates (0.38 vs 0.14 genotypes cleared/person/month, *p* < 0.001). These differences were greatest for persistently-infected subjects in whom neither new infections nor the clearance of old infections could be detected by microscopy (0.71 new infections and 0.73 cleared infections per month using molecular methods vs 0.000 by microscopy, *p* < 0.001).

**Conclusions:**

Molecular methods provide information about genetic diversity, the intensity of transmission and spontaneous clearance in the absence of drug treatment that cannot be obtained by microscopy. They will be necessary to evaluate the efficacy of vaccines, drugs and other control strategies for diseases such as malaria in which simultaneous infection with more than one organism (genotype) is common.

## Background

The epidemiology of infectious diseases is typically based on non-molecular methods, such as microscopy for malaria and other parasites [1], culture for bacteria, fungi and some viruses [2, 3] and serology (including antigen detection) for other bacteria, fungi and viruses [4, 5]. These approaches are based in part on the assumption that simultaneous infection with more than one organism (genotype) of the same species is infrequent or unimportant. Although that assumption may be valid for childhood viral infections such as mumps or measles, there are a number of infectious diseases—such as hepatitis C [6], HIV [7, 8] and Epstein-Barr virus [9]—for which this assumption is incorrect.

In this report, we examine the hypothesis that malaria is one such infectious disease: that simultaneous infection with more than one *Plasmodium falciparum* genotype is common in hyperendemic areas and has potentially important implications for understanding the prevalence, incidence, clearance and control of *P. falciparum* infection in humans. The data reported here demonstrate that simultaneous infection with more than one malaria parasite genotype is common in Mali; they suggest that it may also be common elsewhere in sub-Saharan Africa and in other malarious areas with intense transmission such as Papua New Guinea in the Pacific [10].

## Methods

### Study protocol

Children from 6 months to 9 years of age were identified from the Bancoumana, Mali village census and invited to participate in a prospective study of asymptomatic *P. falciparum* infection. After approval by Institutional Review Boards in Mali, Geneva and New Orleans, this study was presented to the chief, elders, women’s council and parents of the village before obtaining informed consent from the parents and guardians of 80 children from 6 months to 9 years of age. Those 80 children were then followed for 12 months with monthly thick smears and filter paper blots. Children with symptomatic infections were treated free of charge 7 days a week without regard to study participation at the Village Health Centre by a community physician supported by the Village Health Centre or a paediatrician supported by the Mali-Tulane Tropical Medicine Research Center. Symptomatic children were treated according to Ministry of Health guidelines: uncomplicated malaria: oral chloroquine (10 mg base per kg on days 1 and 2, 5 mg base per kg on day 3); Severe or cerebral malaria: intravenous quinine by infusion (8.3 mg base every 8 h initially followed by oral treatment with 8.3 mg base 3 times daily for a total of 7–10 days) and referred to health centers or district hospitals whenever possible. Please note that rapid diagnostic tests (RDTs) were not available or recommended in rural Mali at the time this study was performed. Using this strategy (rapid treatment of symptomatic infection), the frequency of severe disease decreased over a 4 year period from 50–65 to 2–3 cases per year so it became necessary to move studies of severe and cerebral malaria from this village (Bancoumana) to a paediatric referral hospital in the capital city of Bamako (Hôpital Gabriel Touré).

### Thick smears and filter paper blots

The number of asexual parasites per 300 white cells was multiplied by 25 to estimate the number of parasites per μl—based on a white cell count of 7500/μl [11]. Slides for which counts differed by >10 % were re-examined by a senior microscopist to resolve discrepancies.

### Extraction and amplification of parasite DNA

Filter paper blots were extracted as described previously [12–14]. After extraction and storage at 4 °C, parasite DNA was amplified using the polymerase chain reaction (PCR) with *Taq* polymerase and allotype-specific primers for the Block 2 region of *msp1* [12–14]. After electrophoresis and staining with ethidium bromide (0.3 mg/ml), double-stranded DNA fragments (amplicons), which differed in size by ≥10 bp on agarose gel electrophoresis or were produced using different sets of primers, were defined as different parasite genotypes [12].

### Microscopic and molecular definitions of *Plasmodium falciparum* infection

*Infection* A positive thick smear for asexual *P. falciparum* parasites.

*Prevalence* (*based on microscopy*) The fraction (percent) of infected (smear-positive) persons in the group being studied (e.g., children 6 months to 9 years of age).

*Molecular estimate of the number of infections per person* (*molecular analogue of prevalence*) The number of infections (parasite genotypes) detected divided by the number of persons studied.

*Incidence* The risk of acquiring *P. falciparum* infection per unit time—i.e., the number of persons converting from smear-negative (uninfected) to smear-positive per month divided by the number of smear-negative persons at the beginning of the month.

*Molecular incidence* The number of new infections (parasite genotypes) per month divided by the number of persons studied. Because new infections (new parasite genotypes) can be distinguished from existing infections (genotypes), this denominator includes all the children in the cohort (i.e., it also includes the children who were infected at the start of the month).

*Clearance* The fraction of infections cleared per month (i.e., the fraction of smear-positive cases converting to smear-negative) based on spontaneous clearance by the infected individual in the absence of antimalarial drug treatment.

*Molecular clearance* The number of infections (parasite genotypes) cleared spontaneously (in the absence of anti-malarial treatment) per month divided by the number of persons studied. Because the clearance of individual infections (genotypes) can be detected in samples from subjects with multiple parasite genotypes, the denominator for molecular clearance is all infected persons in the cohort (including children who were infected with multiple parasite genotypes at the beginning of the month).

### Management of subjects and data in the study cohort

Children who missed a monthly follow-up visit were re-examined and re-entered into the cohort as soon as possible (usually the following month). Children who had positive blood smears and were symptomatic were treated for malaria free of charge, removed from the cohort for 4 weeks and re-entered into the cohort the following month.

### Statistical analysis

The Chi-square test was used to compare the results obtained with microscopic vs. molecular methods.

## Results

### Prevalence of infection and the number of parasite genotypes per person

**Fig. 1:**
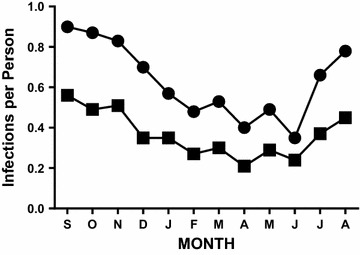
Number of infections per person (prevalence of *P. falciparum* infection). Data provided compare slide-based estimates of the number of infections per person (*filled squares*) with molecular estimates of the number of infections per person (*filled circles*) for the 80 subjects in the pilot cohort

Seasonal patterns for the prevalence of infection (fraction of persons infected) and the number of infections (parasite genotypes) per person were similar using microscopic and molecular methods. However, more infections were detected using molecular methods (571 vs 331 infections in 906 specimens—Table 1) and molecular estimates of the number of infections per person were consistently higher than microscopic estimates (Fig. 1). For example, microscopic estimates of prevalence varied from 0.21 to 0.56 infections per person (positive smears in 21–56 % of the children in the cohort). In contrast, molecular estimates varied from 0.35 to 0.90 infections (parasite genotypes) per person and were greater than microscopic estimates at each time point. Each month, an average of 20 infections (42 % of infections as defined by parasite genotype) were missed by microscopy (ranging from a high of 30 in October [near the peak of the transmission season] to a nadir of eight in June during dry season) (Χ^2^ = 124.45, df = 11, *p* < 0.001).

**Table 1:** Molecular and microscopic estimates of the transmission and dynamics of human *Plasmodium falciparum* infection

	Molecular criteria	Microscopic criteria	Molecular:microscopic ratio
Infections per person (equivalent to prevalence for microscopy)	0.63 (571/906)	0.37 (331/906)	1.73
Incidence (new infections per person per month)	0.38 (311/829)	0.13 (104/829)	2.99
Clearance (infections cleared per person per month)	0.38 (317/829)	0.14 (116/829)	2.73

### Incidence

**Fig. 2:**
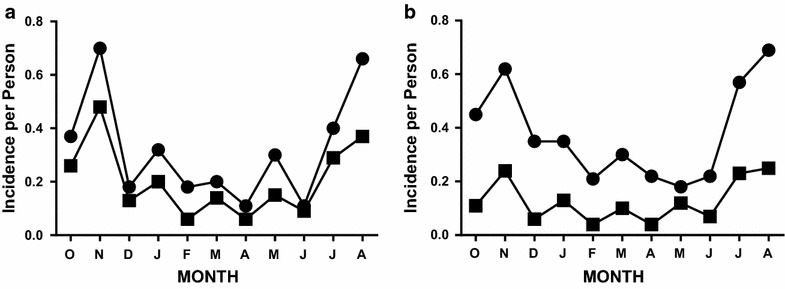
Incidence of *P. falciparum* infection. **a** Slide-negative subjects. Data compare slide-based estimates of incidence (*filled squares*) with molecular estimates of incidence (*filled circles*) for the subset of subjects who were slide-negative at the beginning of each monthly observation period. **b** All subjects in the cohort. Data compare slide-based estimates of incidence (*filled squares*) with molecular estimates of incidence (*filled circles*) for all subjects in the cohort

As with prevalence, the seasonal patterns for incidence were similar with both microscopic and molecular methods (Fig. 2a, b). However, three times as many new infections were detected using molecular methods (311 vs 104 new infections during the 829 person-months of observation—Table 1). The initial comparison of molecular and microscopic incidence was based on subjects with negative smears at the beginning of the observation period, who could therefore be followed for the acquisition of infection by both methods (Fig. 2a). That comparison demonstrated a greater incidence of infection using molecular methods [an average of five new infections per month were missed by microscopy with a peak of 9 [(September to October) and a nadir of 1 (May to June), Χ^2^ = 39.64, *p* < 0.001]. The difference between molecular and microscopic incidence was greater for the cohort as a whole because it was possible to detect new infections using microscopy only in subjects who were smear-negative at the beginning of the observation period. Thus, for the cohort as a whole, an average of 17 new infections per month were missed using microscopy: 207 of 311 new infections (67 %) were missed during the 12 month study period with a peak of 29 [for October to November near the peak of the transmission season] and a nadir of 0 (during the dry season from April to May), Fig. 2b, Χ^2^ > 100, *p* < 0.001.

### Clearance

**Fig. 3:**
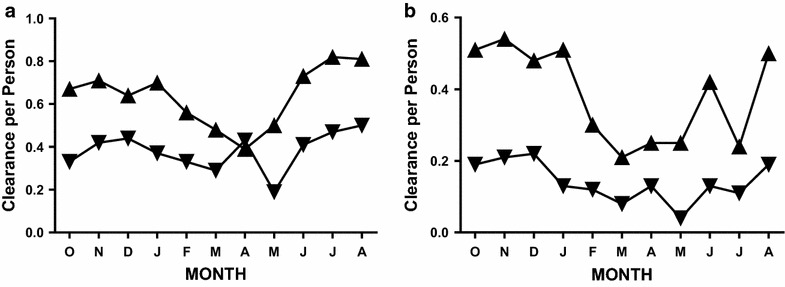
Spontaneous clearance of *P. falciparum* infection. **a** Slide-positive subjects. Data compare slide-based estimates of clearance (*inverted triangles*) with molecular estimates of clearance (*triangles*) for subjects who were slide-positive at the beginning of each monthly observation period. **b** All subjects in the cohort. Data compare slide-based estimates of clearance for all subjects (*inverted triangles*) with molecular estimates of clearance (*triangles*) for all the subjects in the pilot cohort

As with prevalence and incidence, seasonal patterns for clearance were similar with both microscopic and molecular methods (Fig. 3a, b). The initial comparison of molecular and microscopic clearance was restricted to subjects with positive smears at the beginning of the month and negative smears at the end of the month (Fig. 3a) because clearance can be detected by microscopy only in subjects who are smear-negative (uninfected) at the end of the observation period. Based on these criteria, an average of 7 clearance events was missed each month by microscopy [with a peak of 15 (September to October), and a nadir of 0 (March to April), Χ^2^ = 64.28, *p* < 0.001]. In contrast, for the cohort as a whole (Fig. 3b), an average of 17 clearance events per month were missed by microscopy: 201 clearance events were missed during the 12 month study period with a peak of 29 (December to January at the end of the transmission season) and a nadir of 9 (during the dry season from March to April and June to July) (Fig. 3b, Χ^2^ > 100, *p* < 0.001).

### Persistently-infected subjects

**Fig. 4:**
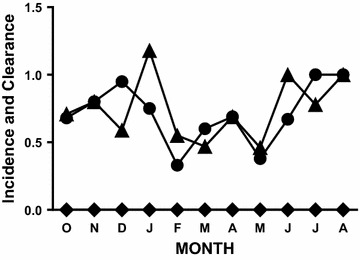
Incidence and clearance of *P. falciparum* infection in persistently-infected subjects. Data compare molecular estimates of incidence (*filled circles*) and molecular estimates of clearance (*filled triangles*) with microscopic estimates of incidence and clearance for subjects who were slide-positive at the beginning and the end of each monthly observation period (*diamonds*)

Subjects with *P. falciparum* parasites at both the beginning and the end of the observation period presented a special challenge because neither the incidence of new infections nor the clearance of old infections could be detected in those subjects by microscopy. The molecular results demonstrate that persistently-infected (persistently smear-positive) subjects were at risk of new infections and often cleared one or more parasite genotypes during the monthly observation periods (Fig. 4). The magnitudes of these discrepancies were substantial: 127 new infections and 131 cleared infections were identified during 179 person-months of observation in persistently-infected subjects using molecular methods, none of which could be identified by microscopy (Fig. 4). Thus an average of 11 new parasite genotypes per month (incidence events) and 11 clearance events per month were missed by microscopy [with peak discrepancies of 21 for incidence (November–December at the end of the transmission season) and 20 for clearance (September–October and December–January); the smallest discrepancies for incidence and clearance were observed during the dry season in April and May, and were 5 and 6, respectively].

## Discussion

### Simultaneous infection with multiple parasite genotypes

The data reported here demonstrate that simultaneous infection with more than one parasite genotype can be detected with molecular methods, although the Block 2 PCR yields a conservative (minimal) estimate of the number of parasite genotypes in a specimen because it examines only a single polymorphic locus in the parasite genome [13, 15, 16]. Thus the genetic diversity identified in these studies would have been greater if additional molecular markers had been used—e.g., merozoite surface protein 2 (*msp2*) [17], circumsporozoite protein (*csp*) [18], glutamate-rich protein (*glurp*) [19] or histidine-rich protein 2 (*hrp2*) [20] in addition to the Block 2 region of *msp1*—or alternative methods such as microarrays or whole genome sequencing.

The genetic diversity identified in these studies reflects the dynamics of transmission and infection in Mali. For example, the molecular:microscopic ratios (Table 1) can be viewed as reflecting: (1) the genetic diversity of the parasite population infecting humans (mean number of parasite genotypes per person), (2) transmission intensity (incidence) and (3) the efficacy of the host immune response in vivo (spontaneous clearance). These interpretations of the prevalence and incidence data are consistent with the seasonal variation observed in the molecular:microscopic ratio for incidence. Thus, the molecular:microscopic ratios for prevalence and incidence were greatest at the end of the rainy season in October and November when transmission was most intense (followed closely by the peak of clearance in December–January), and lowest in May when transmission was undetectable.

This interpretation of the data is likewise consistent with the report of Robert et al. on *P. falciparum* in Senegal [21] and the observations of Kolakovich et al. on *P. vivax* in Papua New Guinea [22]. Although those reports did not discuss the epidemiologic significance of simultaneous infection with multiple parasite genotypes, their findings and these observations raise questions about the effects of genetic diversity on the epidemiology, transmission and persistence of malaria parasites. In addition, the report of Mueller et al. [10] raises the questions noted above about the potential effects of parasite diversity on the epidemiology, transmission and human impact of *P. falciparum* malaria. Those questions, which are potentially relevant to other infectious diseases, include: (1) the impact of simultaneous infection with multiple organisms (genotypes) of the same species on traditional epidemiologic parameters such as prevalence, incidence and clearance and the risk of disease, (2) the intensity of transmission, (3) the incidence and clearance of infection in persistently-infected subjects, (4) the evaluation of candidate vaccines and anti-malarial (and other anti-infective) drugs and (5) the relevance of these observations for other infectious diseases in which simultaneous infection with multiple genotypes is common—such as hepatitis C [6], HIV [7, 8] and hepatitis C [9].

### Epidemiologic parameters

Molecular estimates of epidemiologic parameters (number of infections per person, incidence, clearance) were consistently higher than microscopic estimates (Table 1; Figs. 1, 2, 3 and 4). The additional transmission and clearance events identified with molecular methods provide information that cannot be obtained with non-molecular methods (microscopy). Please note that the estimated incidence of new infections/genotypes in Mali (311 per 829 person-months of observation = 4.50 infections per person-year) is remarkably similar to the estimate of 5.9 infections per person-year recently reported from Papua New Guinea [10].

### Transmission during the dry season

Previously, it has been assumed that the transmission of *P. falciparum* infection to humans was unmeasurably low during the dry season in countries such as Mali. The major reasons for this assumption were that it had been difficult to find anopheline mosquitoes during the dry season and even more difficult to find anophelines infected with *P. falciparum* parasites. In contrast, the data presented here (filled circles, Fig. 2a, b) demonstrate that significant transmission occurs during the dry season between December and May. Despite the almost complete absence of rainfall and undetectably low numbers of anophelines, the incidence of new infections (new parasite genotypes) during the dry season ranged from 0.18 to 0.35 new infections per person per month. This evidence for dry season transmission (incidence of infection with new parasite genotypes) occurs at a time when few or no vectors can be found and is therefore consistent with the mark-release-recapture results reported recently from Bancoumana [23], which likely explain this dry season transmission.

### Persistently-parasitaemic subjects

Based on microscopy alone, one would conclude there were no significant transmission (incidence) or clearance events among children who were parasitaemic (smear-positive) at both the beginning and the end of an observation period. In contrast, using molecular methods, it was possible to identify new parasite genotypes (incidence events), to demonstrate the clearance of old infections among these children and thus to establish that these children actually had a higher incidence of new infections and more frequent clearance of old infections than the rest of the study cohort (Fig. 4 vs Figs. 2, 3).

### Spontaneous clearance

The clearance of *P. falciparum* infection in the absence of antimalarial treatment has been described previously [24] and in subjects given malaria for the treatment of syphilis [25]. However, its frequency under field conditions has been unclear. The results reported here indicate that clearance of *P. falciparum* infection is a frequent event in Mali and thus suggest that clearance of infection in the absence of anti-malarial treatment (spontaneous clearance) is an important aspect of human infection under field conditions. The clearance rate estimated from microscopy in these studies (0.14 infections per person per month—Table 1) is remarkably similar to the clearance (recovery) rate reported previously from the Garki Project in Nigeria 40 years ago (0.06–0.15 infections cleared per person per month) [26].

### Alternative approaches to the evaluation of vaccines

Infection with homologous vs heterologous organisms after immunization and the spontaneous clearance of infection in the absence of treatment. The traditional approach to the evaluation of vaccine efficacy is to compare the incidence of infection among immunized subjects vs. unimmunized controls. The molecular strategies outlined in this report provide two potentially complementary approaches to the evaluation of vaccines: (1) differences in the incidence of infection with homologous vs heterologous parasites after immunization and (2) changes in clearance rates (in the absence of antimalarial treatment) after immunization. To illustrate the first, we have previously compared the incidence of infection with homologous vs heterologous parasites after immunization with SPf66 as a proxy for vaccine efficacy [14]. Those results demonstrated that molecular studies on relatively small numbers of subjects identified a lack of protection against infection with heterologous parasites and thus provided a preliminary assessment of vaccine efficacy more rapidly and more economically than the larger studies which must be employed if severe disease or other infrequent clinical events are the endpoints. The second alternative approach is based on the frequency of spontaneous clearance. In diseases such as malaria (in which repetitive natural infection does not produce sterile immunity), increases in spontaneous clearance may be as important as reductions in the incidence of new infections as a measure of vaccine efficacy and should be considered in the evaluation of candidate vaccines.

### Evaluation of candidate anti-malarial drugs

In the endemic areas where anti-malarials are evaluated for efficacy, interpretation of the results may be confounded by new infections acquired after treatment that appear before or at the times of the 4–6 week (28–42 day) follow-ups. Molecular methods such as the Block 2 PCR used in this study provide simple strategies to determine whether the parasites obtained at follow-up are different from those present at the time of initial diagnosis and thus to distinguish drug failure (recurrent infection with the same parasite genotypes) [27] from successful treatment of the initial infection followed by reinfection (new infections with different parasite genotypes) which do not indicate treatment failure.

### Generalizability

The results reported here demonstrate that molecular methods provide a dynamic perspective on the transmission and clearance of *P. falciparum* infection that cannot be obtained with microscopy. Although these studies examined *P. falciparum* malaria, infection with multiple organisms/genotypes of the same species is not restricted to malaria. Thus the lessons learned from these studies are potentially applicable to a broad range of infectious diseases; they should encourage re-examination of the traditional parameters used to assess the epidemiology of infectious diseases (prevalence, incidence, clearance) and the evaluation of candidate interventions (vaccines, drugs) for control of those infectious diseases. Simultaneous infection with more than one genotype is also characteristic of infections due to microbes very different from *P. falciparum*, such as hepatitis C [6], HIV [7, 8] and the Epstein-Barr viruses [9]. However, there has been little discussion about the epidemiologic implications of those observations or their potential relevance for the study of candidate vaccines and drugs.

### Potential limitations

Potential limitations of these studies include the fact that no tests (including the molecular approached used here) are 100 % sensitive; there are always some false-negatives. In addition, the modeling studies reported by Bretscher et al. suggest that false-negative readings at a single point in time may lead to overestimation of the frequency (rate) of parasite clearance [28]. In these studies, that concern was addressed by requiring that parasite genotypes be absent for a minimum of 2 consecutive monthly collections before they were classified as clearance events.

## Abbreviations

*Csp*: Circumsporozoite protein
*Glurp*: GLUtamate-rich protein
*hrp2*: Histidine-rich protein 2
*msp1*: Merozoite surface protein 1
*msp2*: Merozoite surface protein 2
PCR: Polymerase chain reaction
